# Assessing “First Mile” Supply Chain Factors Affecting Timeliness of School-Based Deworming Interventions: Supply and Logistics Performance Indicators

**DOI:** 10.1371/journal.pntd.0004115

**Published:** 2015-12-10

**Authors:** Kimberly M. Koporc, Eric Strunz, Cassandra Holloway, David G. Addiss, William Lin

**Affiliations:** 1 Children Without Worms, Task Force for Global Health, Decatur, Georgia, United States of America; 2 Johnson & Johnson, New Brunswick, New Jersey, United States of America; Ministère de la Santé Publique et de la Lutte contre les Endémies, NIGER

## Abstract

**Background:**

Between 2007 and 2012, Children Without Worms (CWW) oversaw the Johnson & Johnson (J&J) donation of Vermox (mebendazole) for treatment of school-age children to control soil-transmitted helminthiasis (STH). To identify factors associated with on-time, delayed, or missed mass drug administration (MDA) interventions, and explore possible indicators for supply chain performance for drug donation programs, we reviewed program data for the 14 STH-endemic countries CWW supported during 2007–2012.

**Methodology:**

Data from drug applications, shipping records, and annual treatment reports were tracked using Microsoft Excel. Qualitative data from interviews with key personnel were used to provide additional context on the causes of delayed or missed MDAs. Four possible contributory factors to delayed or missed MDAs were considered: production, shipping, customs clearance, and miscellaneous in-country issues. Coverage rates were calculated by dividing the number of treatments administered by the number of children targeted during the MDA.

**Principal Findings:**

Of the approved requests for 78 MDAs, 54 MDAs (69%) were successfully implemented during or before the scheduled month. Ten MDAs (13%) were classified as delayed; seven of these were delayed by one month or less. An additional 14 MDAs (18%) were classified as missed. For the 64 on-time or delayed MDAs, the mean coverage was approximately 88%.

**Conclusions and Significance:**

To continue to assess the supply chain processes and identify areas for improvement, we identified four indicators or metrics for supply chain performance that can be applied across all neglected tropical disease (NTD) drug donation programs: (1) donor having available inventory to satisfy the country request for donation; (2) donor shipping the approved number of doses; (3) shipment arriving at the Central Medical Stores one month in advance of the scheduled MDA date; and (4) country programs implementing the MDA as scheduled.

## Introduction

In 2016, pharmaceutical companies will ship more than 1,000 million doses of medicine to support mass drug administration (MDA) interventions in Africa, Asia, and Latin America and the Caribbean to control or eliminate blinding trachoma, lymphatic filariasis, onchocerciasis, schistosomiasis, and soil-transmitted helminthiasis (STH), all of which are neglected tropical diseases (NTDs) because they affect the poorest of the poor and often the hardest to reach populations [1]. Missed MDAs negatively impact control and elimination [2] initiatives and waste valuable resources. Little is known about the timeliness of MDAs or factors associated with on-time, delayed, or missed MDAs.

**Fig 1:**
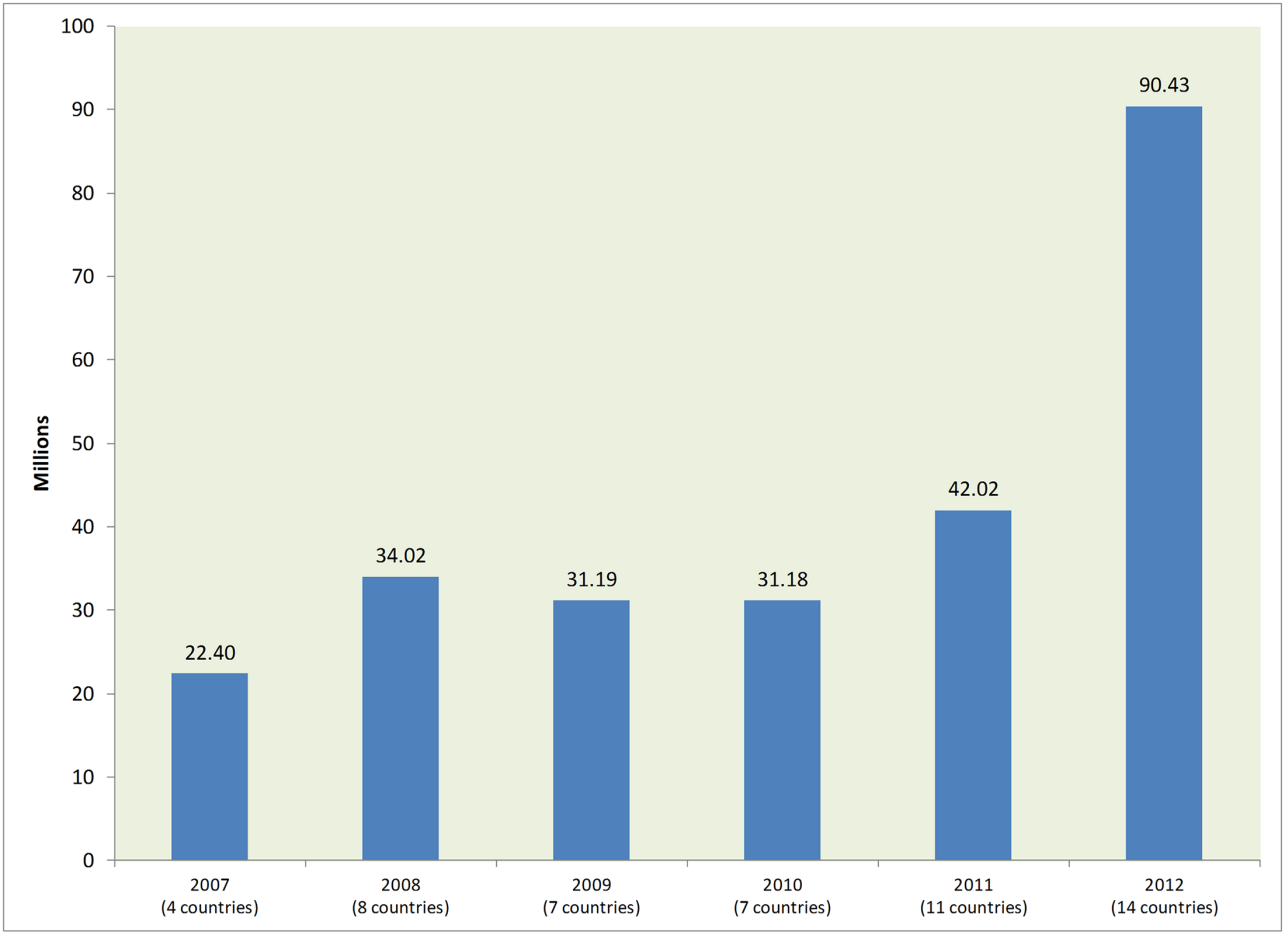
Approved Vermox doses (millions) and number of countries supported by year.

Since 2007, Johnson & Johnson (J&J) has donated Vermox (mebendazole) for treatment and control of STH in school-age children. Between 2007 and 2012, national ministries of health or education requested Vermox through Children Without Worms (CWW), which facilitated drug shipments and provided technical support for STH control. The donation scaled up in 2008 when the number of countries receiving Vermox increased from four to eight. For most of these years, seven to eight countries received 30–33 million doses of Vermox. These figures increased substantially in 2011 and 2012 when the number of countries receiving the donation increased again (Fig 1).

From 2007–2012, countries requested Vermox by submitting applications to CWW. Applications included information on disease burden, number of school-age children targeted for treatment, date of scheduled MDAs, and mechanisms for distributing Vermox. The applications also included strategies for monitoring and evaluation, providing appropriate training and supervision to teachers and health workers responsible for distributing medicine, and preventing reinfection (i.e., provision of health education, water, and sanitation). Once the applications were complete, CWW submitted them to its advisory committee for review and approval. CWW then worked with J&J to schedule production and shipments of the Vermox so that they arrived in time for the scheduled MDAs. Once consignments arrived in country and cleared customs, they were transported to government Central Medical Stores (CMS), which were responsible for organizing transportation of the donated medicines to endemic communities.

To identify factors associated with on-time, delayed, or missed MDAs and explore possible indicators for supply chain performance for NTD programs, CWW reviewed program data on Vermox requests, production, shipments, and treatments for the 14 STH-endemic countries it supported during 2007–2012.

## Methods

### Sources of data

Three sources of data were used for the analysis: (1) drug applications, (2) shipping records, and (3) annual treatment reports. Data regarding donations to countries (e.g., doses approved, doses shipped, shipping dates, and dates doses were distributed) were tracked by CWW using Microsoft Excel. Qualitative information was collected via interviews with key personnel to provide additional context on the causes of delayed or missed MDAs. Table 1 summarizes the information collected for analysis of this supply chain performance.

**Table 1:** Collection and use of supply chain information.

Information Collected	Source	Information Used To:
1. Doses approved	Approval Letter—This document confirmed the number of treatments approved, the number of annual MDAs planned, and the number of tablets to be shipped.	• Determine the number of doses to be shipped.
2. Month of scheduled MDA	Application—This document included the month(s) of the scheduled MDAs, the number of children to be treated, and the targeted districts.	• Determine the date the consignment must be shipped (typically 3 months prior to MDA if by sea or 1 month prior to MDA if by air).
3. Date consignment shipped	Shipping Notice—This document was sent by J&J to the consignee informing him or her that the consignment is on its way. Copies of the shipping documents are included with the notice.	• Determine whether the consignment shipped on time
		• Calculate the number of days it took to transport the consignment to the port of entry
4. Date consignment arrives in port of recipient country	Various sources: DHL, the company contracted by J&J to organize consignments; Consignee; and Receiving Report	• Calculate the number of days it took to transport the consignment to the port of entry
	The receiving report, completed by the consignee, confirmed receipt of donation and included the date consignment was received and number and condition of tablets received by the government CMS.	• Calculate the number of days it took the consignment to clear customs
5. Date consignment cleared customs	Various sources: DHL; Consignee (CWW generally did not collect information unless there was a problem)	• Calculate the number of days it took the consignment to clear customs
6. Month MDA was implemented	Annual Treatment Report—This document provided the month(s) the MDA was implemented and number of children treated in each district. This report was submitted to WHO Headquarters by CWW for inclusion in the Preventive Chemotherapy Databank.	• Compare month of implemented MDA with month of scheduled MDA
7. Number of children treated	Annual Treatment Report	• Assess treatment coverage rates

### Timeliness of MDA

On-time, delayed, and missed MDAs are defined as follows:

- On-time MDA—an MDA was defined as on-time if it was implemented during the scheduled month.
- Delayed MDA—an MDA was defined as delayed if it started after but within six months of the originally scheduled month.
- Missed MDA—an MDA was defined as “missed” if it started more than six months after the originally scheduled month (see definition of delayed MDA).

The unit of measurement for this analysis was calendar months. The scheduled month of an MDA was indicated by countries in their annual application to CWW. The implementation month of the MDA was indicated by countries in their annual treatment report. Because only the months were reported for the scheduled and implemented MDAs (i.e., dates were not specified), the analysis assumes that the scheduled MDA would have occurred on the first of the month and the implemented MDA occurred on the last day of the month. For example, if the MDA was scheduled to take place in May but was implemented in June, we assumed that the MDA was scheduled to start May 1 but was implemented June 30—two months later.

### Contributory factors to delayed or missed MDAs

For delayed or missed MDAs, we considered four possible contributory factors. For the purpose of our analysis, delayed and missed MDAs were attributed to the first-listed cause in the supply chain process, even if subsequent factors were contributory. Secondary factors contributing to delays were also noted and analyzed.

#### Production

Production was considered on time if the consignment was produced and prepared for shipment by the production facility three months (if by sea) or one month (if by air) prior to the scheduled MDA. Three months if by sea and one month if by air are metrics that CWW and J&J used since the inception of the program. There were no requests from countries for rush orders between 2007 and 2012. Therefore, these metrics are suitable for all shipments considered in this analysis. If a production problem resulted in a delay that rippled throughout the supply chain process and ultimately impacted a scheduled MDA, the primary cause of delay was attributed to production.

The unit of measurement for this analysis was calendar months. Because the specific date of the MDA was not available, the length of time between the shipping event and the MDA was rounded up or down to the first day of the month to yield the most conservative estimate. For example, if a consignment was sent on January 15th and the MDA was scheduled for some time in March, for the purposes of the analysis, the shipment date was rounded up to February 1 and the MDA date was assumed to be March 1 to yield an interval of only one month. We used the Microsoft Excel function DATEDIF to calculate the time intervals.

#### Shipping

If there were no production issues and the shipment departed from the production facility on time and the consignment did not arrive one month prior to the date of the scheduled MDA, the primary cause of delay was attributed to shipping.

#### Customs clearance

If there were no production or shipping issues, and if the consignment was held up in customs two weeks or more (usually the point that it began to incur demurrage), then the primary cause of the delay was attributed to problems with customs clearance.

#### Miscellaneous in-country delay

If an MDA was missed or delayed and there were no production, shipping, or customs issues, then the primary cause was attributed to miscellaneous factors within the country (e.g., weather, scheduling conflicts, lack of resources and/or coordination, or in-country transportation issues).

### Treatment coverage

Coverage rates, expressed as a percentage, are calculated by dividing the number of treatments administered by the number of children targeted during the MDA.

## Results

An independent Advisory Committee, for which CWW serves as the secretariat, approved requests for 78 MDAs in 14 countries between 2007 and 2012. Fifty-four MDAs (69%) were successfully implemented during or before the scheduled month. Ten MDAs (13%) were classified as delayed; seven of these were delayed by one month or less. An additional 14 MDAs (18%) were classified as missed.

### Delayed MDAs

Table 2 summarizes the factors contributing to the ten delayed MDAs. Seven of the ten delayed MDAs were experienced by one country, all of those seven were attributed to in-country issues, and six of them lasted only one month. Unlike countries with larger populations, this country had the flexibility to vary the month of their MDAs from year to year, and so these “delays” caused minimal or no disruption in the STH control program.

**Table 2:** Delayed MDAs, 2007–2012.

Country	Year	Round	Delay Length (Months)	Doses Approved	Primary Cause of Delay	Secondary Cause of Delay
A	2007	1	1	4,000,000	Shipping	Customs
B	2008	1	1	85,000	Shipping	Customs
B	2008	2	2	85,000	In-Country	None
B	2009	1	1	90,000	In-Country	None
B	2009	2	1	90,000	In-Country	None
B	2010	1	1	90,000	In-Country	None
B	2010	2	1	90,000	In-Country	None
B	2011	1	1	90,000	In-Country	None
C	2012	1	3	1,900,000	In-Country	None
D	2012	2	2	300,000	In-Country	None

There were two delays attributed to shipping and further aggravated by customs clearance issues. The consignment of the first delay in 2007 was sent by sea less than the desired three months in advance of the scheduled MDA. The consignment of the second delay was in 2008 and was shipped by air less than a full month in advance of the scheduled MDA.

### Missed MDAs

Table 3 summarizes the factors contributing to the 14 missed MDAs. The cause of six missed MDAs was attributed to miscellaneous in-country issues. Five missed MDAs were caused by challenges in production, and three missed MDAs were caused by challenges in customs clearance.

**Table 3:** Missed MDAs, 2007–2012.

Country	Year	Round	Doses Approved	Cause of Missed MDA
E	2008	1	6,000,000	Production
F	2008	1	4,000,000	Production
A	2008	2	4,000,000	Customs
G	2008	1	1,500,000	In-Country
B	2011	2	90,000	In-Country
H	2011	1	1,760,000	Production
D	2011	1	300,000	Production
C	2011	1	1,922,305	Production
I	2012	1	5,500,000	In-Country
I	2012	2	1,500,000	In-Country
H	2012	1	4,750,000	Customs
H	2012	2	1,650,000	Customs
C	2012	2	1,900,000	In-Country
G	2012	1	1,200,000	In-Country

### Treatment coverage

**Fig 2:**
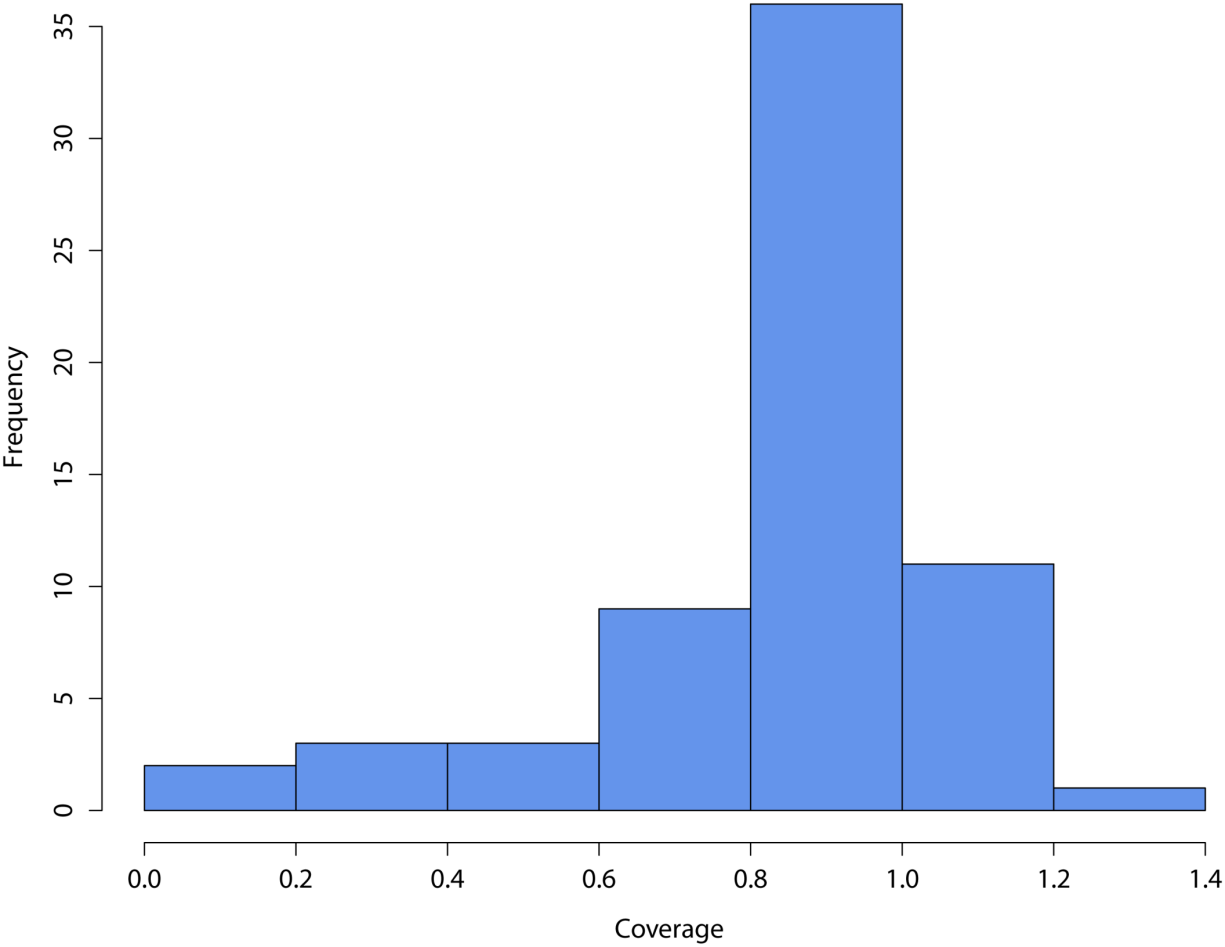
Distribution of reported treatment coverage, 64 CWW-supported MDAs, 2007–2012.

For the 64 MDAs that were implemented between 2007 and 2012, the mean coverage was approximately 88% with a median of 94%. Fig 2 shows the distribution of treatment coverage. Thirty-six of the 64 MDAs (55%) achieved more than 90% of their coverage targets. The lowest coverage was 7%, and the highest was 123%. For 12 MDAs, reported coverage exceeded 100%. For nine of these 12 MDAs, coverage was less than 108% and was attributed to one or more of the following factors: rounding error, use of remaining stock to treat other populations, or inclusion of treatment data with benzimidazoles from other sources. For the MDAs that had coverage rates of 108%, 117%, and 123%, extra tablets were extended to treat teachers and health workers as an incentive, or the target population was expanded to other districts. In the latter case, the target population (the denominator) had not been adjusted in the annual treatment report to reflect targeting children from other districts. Reported treatment coverage rates were comparable between on-time and delayed MDAs (86% versus 77%, *p* > 0.44).

## Discussion

More than 78% of the CWW-supported MDAs between 2007 and 2012 (61 of 78) occurred on schedule or with a delay of one month or less. Seventeen MDAs were missed or significantly delayed between 2008 and 2012. Analysis indicated that the primary causes were miscellaneous in-country issues and production challenges, which coincided with periods of rapid scale-up. Because delayed or missed MDAs negatively impact control and elimination initiatives and create additional burden for endemic countries, CWW and J&J responded by modifying application and supply chain processes to improve performance.

### Production challenges and solutions

Two of the four missed MDAs in 2008 can be principally attributed to rapid scale-up that challenged drug production. In response, CWW changed the timing of the application process. Before 2008, CWW requested applications from ministries of health or education during the same year of the scheduled MDA. Following the production delays experienced in 2008, CWW began requesting applications a year in advance of the scheduled MDA. In addition, following the 2012 issues regarding production, J&J created a buffer stock.

### Shipping challenges and solutions

Examples of shipping issues include difficulty booking a vessel or delays in organizing secondary transport overland to landlocked countries. For example, shipments to Uganda had to be shipped to Mombasa, Kenya, and then transported overland to Kampala, Uganda. Because the consignment had to travel to two countries and clear two customs agencies, potential bottlenecks were doubled. Using historical records, CWW tracked the number of days consignments took to clear each step (e.g., number of days to arrive in port, number of days to clear customs at first port of entry, and number of days to be placed on a truck and transported overland) and documented causes of shipping delays. CWW used this information to identify and prevent potential bottlenecks with future consignments. For example, if a consignment was taking too long (compared to previous consignments) to be loaded on a ship or arrive at port, CWW contacted the shipping agency, DHL, to investigate and troubleshoot issues.

### Customs clearance challenges and solutions

Although only four of the 24 delayed and missed MDAs were attributed to customs issues, the frequency with which countries experienced challenges with customs was much higher. The efficiency of clearing consignments through customs varied by country; some countries took less than a week and others more than a month. Factors that affected the customs-clearance process were as follows:

1. Changes in customs-clearance requirements that required additional paperwork and processing.
2. The consignee was not familiar with the customs-clearance process.
3. Programs did not have sufficient budget for costs incurred in clearing the consignment and transporting it to CMS. In CWW’s experience, this issue was sometimes exacerbated if the shipping containers were large or refrigerated. These containers require special handling and therefore incur added costs.
4. Landlocked countries pose additional challenges that may include customs clearance processing in multiple countries and overland transport. For example, Uganda’s consignments had to be cleared twice, which adds time and money as well as paperwork.

To avoid delays due to customs, CWW implemented a “green-light” process whereby the consignee confirms that all necessary paperwork required for customs clearance are in order before a consignment is loaded on a vessel. For the donation of Vermox, the critical documents for customs clearance include invoice, packing list, certificate of origin, certificate of analysis, certification of donation, and bill of lading or airway bill. CWW staff also started tracking the shipment until it arrived in CMS. On arrival, CWW alerted the consignee that the consignment arrived at the port and continued monitoring the process until the consignment cleared customs. CWW only started to systematically track customs issues between shipments arriving at the port and final clearance in 2011. Therefore it is not possible to unequivocally state that rigorous monitoring resulted in improvements. However, our overall experience is that customs clearance is a highly complex process and there is a wide range of capacities and expertise in the different countries. Therefore, close monitoring allows for timely intervention where necessary. For example, for those countries that take more than a month to clear consignments through customs, consignments should be shipped at least five months prior to the MDA.

### Transition to World Health Organization

In 2013, oversight for the donation of Vermox was transferred to World Health Organization (WHO) to streamline application processes for many of the medicines donated for the preventive chemotherapy (PC) NTDs. After this transfer, CWW continued to liaise with J&J and WHO on fulfilling the requests for medicines. The continued engagement of CWW in the process ensured a smooth transition. The one important change is that the WHO Country Representative (WR) is the consignee for all consignments. This change has two benefits. First, the WR has staff trained and licensed to clear consignments from customs. And second, WHO is exempt from paying import duties.

## Conclusion

The supply chain lessons learned from the experiences of CWW are relevant to other donation programs because countries are often endemic, with more than one PC NTD. A country’s customs clearance process typically does not change when sources of donation or the organization acting as consignee change. Overall, the cases presented here argue for closer coordination and collaboration between all the players.

### Supply chain performance metrics

In order to continue to assess the supply chain processes and identify areas for improvement, we recommend indicators or metrics for supply chain performance that can be applied across all the donation programs. For the purpose of this analysis, CWW defined “shipping success” as having the following four components:

1. donor having available inventory to satisfy the country request for donation;
2. donor shipping the approved number of doses, at minimum, to requesting countries;
3. in the case of Vermox donation for STH control, shipment arriving at the CMS one month in advance of the scheduled MDA date (shipments need to be dispatched at least three months in advance if sent by sea or one month in advance if sent by air). Lead times for medicines needing to be at the CMS may vary for different NTD programs; and
4. country programs implementing the MDA as scheduled.

Our experience suggests that these four components can serve as the basis for metrics to assess supply chain performance for NTD programs and to measure anticipated improvements resulting from coordinated shipments for MDAs involving multiple drugs for several NTD programs.

### Recommended supply chain bottleneck solutions

CWW and J&J made four changes to the supply chain process to prevent delayed and missed MDAs:

1. the application deadline was moved back to one year prior to the scheduled MDA to facilitate Vermox forecasting and production during years of scale-up;
2. a buffer stock was created to readily respond to increased requests for Vermox;
3. a “green-light” process was initiated to ensure the necessary customs clearance paperwork was completed or close to completion before the consignment was shipped to avoid costly demurrage;
4. CWW proactively tracked shipments to identify and remedy potential bottlenecks.

Implementing the third and fourth changes required a full-time person to identify and develop relationships with key individuals responsible for each step in the process. These relationships enabled CWW and partners to effectively manage unique country-specific issues (e.g., hiring laborers to offload a truck). CWW also provided regular updates to consignees, the ministries, and DHL. CWW documented its supply chain process and shared this document with WHO and other drug donation programs.

**Key Learning Points**

- Understand “first mile” supply chain challenges and identify factors associated with on-time, delayed, or missed mass drug administration interventions.
- Explore possible indicators for supply chain performance of neglected tropical disease control and elimination programs.
- Identify solutions to challenges or bottlenecks in the supply chain process.

**Top Five Papers**

1. Burnham G & Mebrahtu T (2004). The delivery of ivermectin (Mectizan). *Tropical Medicine & International Health* 9 (4): 26–44.
2. World Health Organization (2010). Monitoring drug coverage for preventive chemotherapy. World Health Organization, Geneva.
3. World Health Organization (2015). Investing to overcome the global impact of neglected tropical diseases: third WHO report on neglected tropical diseases. World Health Organization, Geneva.
4. Yadav P, Tata H, Babaley M (2011). The world medicines situation 2011: Storage and supply chain management. World Health Organization, Geneva.
5. Yadav P (2015). Health product supply chains in developing countries: Diagnosis of the root causes of underperformance and an agenda for reform. *Health Systems and Reform* 1 (2): 142–154.
